# What good is maths in studies of wound healing?

**DOI:** 10.1016/j.isci.2022.104778

**Published:** 2022-07-19

**Authors:** Jake Turley, Isaac V. Chenchiah, Tanniemola B. Liverpool, Helen Weavers, Paul Martin

**Affiliations:** 1 School of Mathematics, Fry Building, University of Bristol, Bristol BS8 1UG, UK; 2 School of Biochemistry, University of Bristol, Bristol BS8 1TD, UK

**Keywords:** Computer modeling, Mathematical biosciences

## Abstract

Wound healing is an aspect of normal physiology that we all take for granted until it goes wrong, such as, for example, the scarring that results from a severe burn, or those patients who suffer from debilitating chronic wounds that fail to heal. Ever since wound repair research began as a discipline, clinicians and basic scientists have collaborated to try and understand the cell and molecular mechanisms that underpin healthy repair in the hope that this will reveal clues for the therapeutic treatment of pathological healing. In recent decades mathematicians and physicists have begun to join in with this important challenge. Here we describe examples of how mathematical modeling married to biological experimentation has provided insights that biology alone could not fathom. To date, these studies have largely focused on wound re-epithelialization and inflammation, but we also discuss other components of wound healing that might be ripe for similar interdisciplinary approaches.

## Highlights

• Mathematics can often decipher cell behaviors that biology alone cannot
• Some aspects of wound healing are currently tractable to mathematical approaches
• Appropriate maths approaches range from Bayesian Inference to Deep Learning
• Wound biology provides opportunities to discover and develop new mathematics

## Graphical abstract

**Figure:**
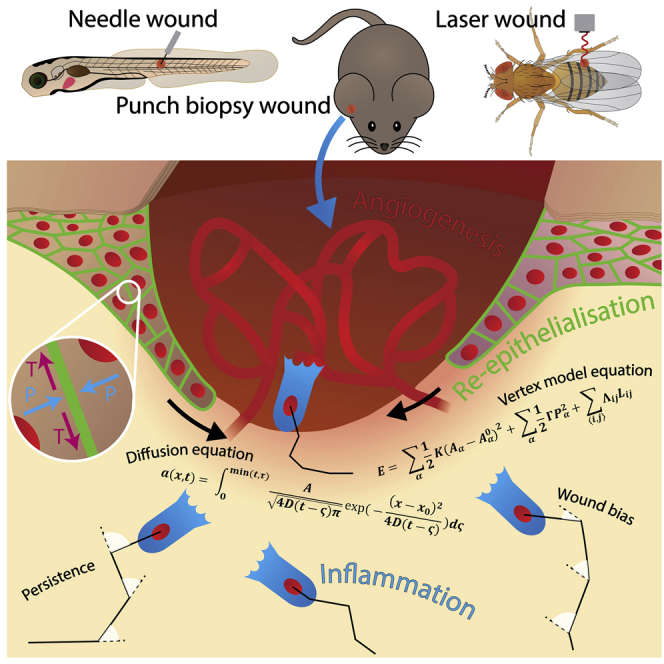

## Introduction

All multicellular organisms have evolved strategies for healing wounds that they may receive by accident or in combat. Generally, these tissue repair mechanisms work very efficiently, often replacing missing tissues with a near-perfect equivalent of what was lost, with minimal scarring. However, in human adults, tissue repair is less perfect than in simpler organisms, and there are occasions and individuals where healing is considerably less than perfect, leading to excessive scarring at the one extreme or a complete failure to heal—resulting in a chronic wound—at the other extreme (Eming et al., 2014).

**Figure 1:**
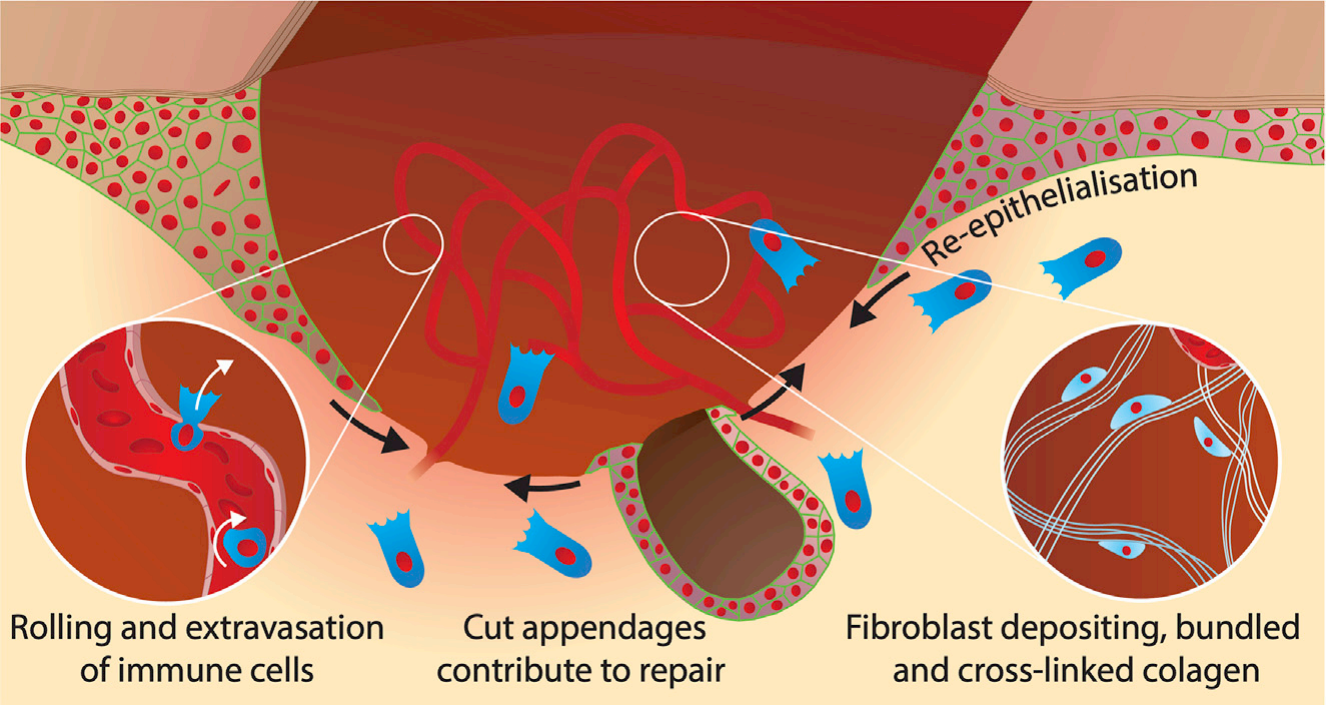
Schematic of a healing wound A schematic of a healing wound. Epithelial cells migrate down between scab (brown) and healthy granulation tissue (yellow) to seal the wound gap. Cut epithelial appendages (eg hair stumps) can contribute to this wound re-epithelialization. Inflammatory cells (dark blue) are drawn to the wound by chemoattractants released from wound edge cells and other “damage” signals. Inflammatory cells roll along the activated luminal surface and then extravasate from vessels adjacent to the wound (left inset), and then orchestrate the activities of many cell lineages at the wound, including the sprouting wound angiogenesis of endothelial cells and the laying down of collagen fibrils by fibroblasts (pale blue) to make wound scar (right inset).

We have learned a great deal about the various cell lineages that contribute to wound healing and much about the signals that drive their behaviors from decades of animal studies in models from *Drosophila* and zebrafish through to mice, as well as a small number of preclinical studies in pigs (Razzell et al., 2011; LeBert and Huttenlocher, 2014; Grose and Werner, 2004; Seaton et al., 2015). In brief, a skin wound—at least in mammals like us—is temporarily plugged with a fibrin clot, and then, over subsequent hours and days (and even weeks), the tissues at the margin grow and move forward to fill in the gap; the superficial epidermal sheet migrates forward to seal the outer barrier layer and, beneath that, a wound granulation tissue assembles from a combination of invading fibroblasts and sprouting blood vessels. Much of what goes on beneath the advancing epidermis is orchestrated by inflammatory cells which are primarily drawn in to fight infection and clear debris, but also double as key signaling hubs directing wound angiogenesis and collagen scar deposition, and well as many of the other cell behaviors that drive wound repair (Figure 1, Box 1, Box 2, Box 3 and Eming et al., 2014).

Box 1 **Wound Inflammation**

The wound inflammatory response is triggered after tissue damage or infection by a variety of damage signals. In vertebrates this inflammatory response is led by neutrophils—some passively leaking from damaged vessels but the majority actively extravasating from intact wound vessels—which kill bacteria by a variety of strategies including ROS release and by “throwing” DNA nets. Neutrophils are followed by macrophages that engulf spent neutrophils and clear other cell and matrix debris, and also orchestrate several other positive episodes required for repair, including wound angiogenesis, but have a negative impact also through their driving of scar collagen deposition. The resolution of inflammatory cells from the wound site is tightly regulated in order to avoid chronic inflammatory pathologies.

Box 2 **Fibroblasts and collagen deposition**

Fibroblasts are recruited into wound granulation tissue to replace missing tissues; a subset of these fibroblasts transform into contractile myofibroblasts which contribute to wound contraction and closure. Wound fibroblasts tend to lay down collagen in cross-linked, oriented bundles, rather than the basket-weave array of unwounded dermis. This scar collagen deposition is “instructed” by wound inflammatory cells and influenced by mechanical tension at the wound site.

Box 3 **Wound angiogenesis**

The reason that healing skin wounds appear pink is the massive sprouting and influx of wound blood vessels to serve the metabolic demands of the healing tissues. They give the new wound connective tissue a “granular” appearance which has led to this tissue being called granulation tissue. Wound angiogenesis is driven by various signals, and largely mediated through VEGF, which is delivered by wound macrophages that also participate in the later resolution of wound vessels after healing is complete.

But there are still many unanswered questions, and for some of these, a marriage of mathematics, physics, and biology studies is likely to deliver where biology alone might not. We are keen to advocate interdisciplinary approaches to the study of key wound healing questions. By this, we don’t just mean quantifying aspects of wound healing or the development of mathematical models that merely simulate what can already be observed in biology. Rather our goal is mathematical modeling that either enables analysis of the often huge and complex datasets that the wound healing community can now generate, to extract functional behaviors and/or modeling which is predictive and guides which biology experiments to do next and thus answers some of the biology questions that would otherwise remain opaque without a robust quantitative framework. Such collaboration between biology, physics, and mathematics is particularly timely because of recent advances in sophisticated imaging techniques which provide so much data that it is now impossible for traditional biology data analysis tools to extract all the details and reveal the otherwise hidden rules of the cell biology of wound healing.

By its very nature, wound healing involves many cell lineages undergoing various behaviors over an extended time-course, and this complexity and the numerous associated biology questions, in turn, generate many interesting and novel mathematical problems. A historically important example of a class of mathematical model inspired by biological systems is the Turing or “reaction-diffusion” model which describes how molecules diffuse and react with one another to spontaneously give rise to complex patterns (Turing, 1990; Kondo and Miura, 2010). These equations are not only fascinating from a mathematical perspective but have been used to understand several aspects of embryonic morphogenesis, for example, the spacing patterns of feathers, or cartilage elements in a developing limb (Painter et al., 2012; Hiscock et al., 2017). Here, for example, mathematical modeling can be used to predict or rule out likely morphogen interactions for any given biological pattern (Kondo and Miura, 2010). However, the new cell-level details that we can now access suggest that such models are not entirely appropriate for describing the processes of wound healing and tissue maintenance. To be specific, these reaction-diffusion models fail to take into account of forces, mechanics, and feedback (i.e., physics) (Bois et al., 2011) that are involved in these tissue responses. However, inspired by the ways in which Turing models have offered such useful insights into our understanding of some aspects of embryonic development, we hope that we can build new, more realistic mathematical models incorporating this application of physics and that similar interdisciplinary mathematics-physics-biology collaborations will benefit wound repair studies also.

## A short “wants” list from the wound community

In the field of wound healing, researchers and clinicians aspire to understand tissue repair sufficiently well to develop interventions that might improve healing in the clinic and allow us to overcome the two extreme wound pathologies of scarring and chronic non-healing wounds. To do this, we need to decipher the full portfolio of cell behaviors involved in wound repair and the ways in which these cells signal to one another. And then we must integrate all of these different cell behaviors together to determine how they drive each of the phases of healing over time, resulting mostly in near-perfect repair of the wound—and how, when elements go awry, this can lead to pathology. Later we discuss some of the wound repair questions that are already being investigated using interdisciplinary maths-biology approaches, as well as some less studied wound processes that also look ripe for the challenge.

### Modeling wound re-epithelialization

The wound community is unsurprisingly enthusiastic to understand how the wound epithelium seals over to cover a wound defect to re-establish the barrier layer, and why sometimes this can fail. Without epidermal sealing chronic wounds are prone to infection, and, as is the case in diabetic foot ulcers, these infections can lead to dire clinical consequences as extreme as limb amputation (Nunan et al., 2014). While it is clear that individual and concerted cell contractions and shape changes, as well as cell movements and cell divisions, must all contribute to the closure of the epithelial hole, how much each of these contributes to the ultimate goal is not known, and neither do we have much idea how any of these cell behaviors might compensate if one or more of the others failed.

**Figure 2:**
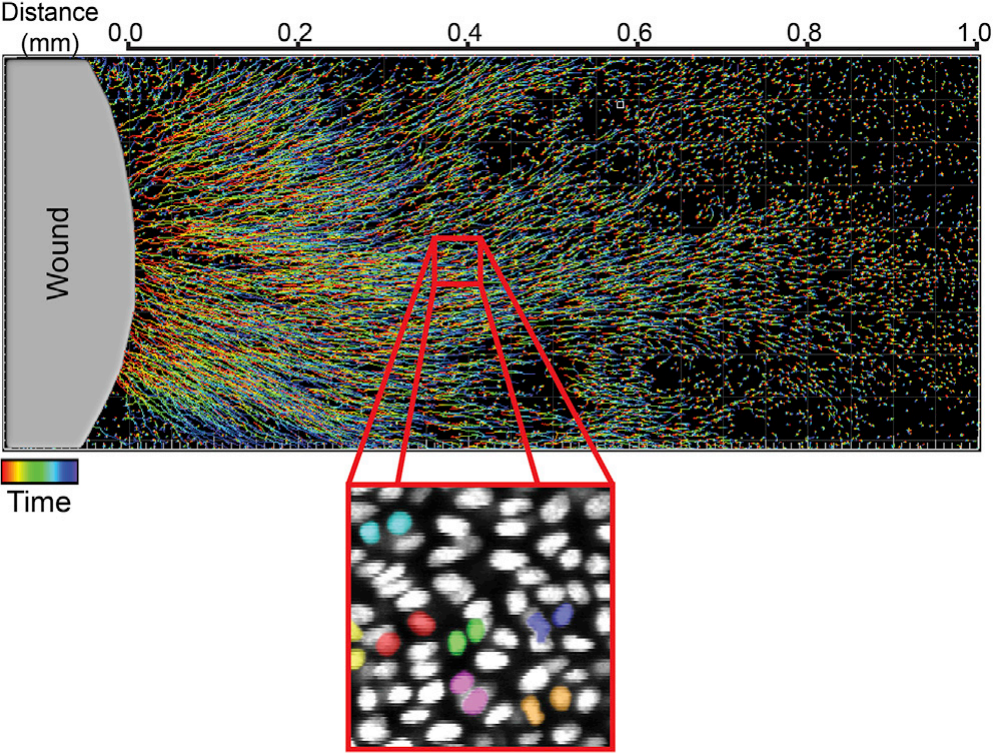
Tracking epithelial cell migration in mouse wound healing Imaging data from the Greco lab show tracks of epithelial cells as they move toward the wound. Cells closer to the wound edge migrate further toward the wound and so have longer tracks. High-resolution nuclear imaging enables the analysis of cell divisions and their orientation bias (Park et al., 2017).

In recent years there have been several elegant live imaging studies of wound re-epithelialization in mouse models using sophisticated state-of-the-art imaging strategies to enable the capture of cell movements and cell divisions within the advancing epidermis. One of these studies targeted wounds made to the tail where the epidermis is devoid of distracting hairs (Aragona et al., 2017). It has long been presumed—but not definitively shown—that the front rows of wound edge cells migrate while those further back proliferate, but this was one of the first *in vivo* studies to show definitive evidence for this. Another study, this time utilizing the thin hairless epidermis of the ear (Park et al., 2017), showed the first high-resolution imaging of an advancing wound epidermis; this too indicated zones of migration at the leading edge and proliferation further back, but suggested that these zones can overlap. This data was of sufficiently high resolution to enable tracking of fluorescently labeled epidermal nuclei and quantification of both migratory speed and proliferative index, and even the possibility to determine the orientation of division in ways that suggest this might be biased toward the wound center (Figure 2 and Park et al., 2017).

Because tissues in mouse and man are opaque, any live imaging approaches will be very technically challenging. Before the studies described above, no lab had been able to directly observe mammalian re-epithelialization *in situ*, and even these pioneering new studies provide rather limited volumes of data, with less than optimal spatial and temporal resolution. The ways to gain higher resolution imaging of wound re-epithelialization are either to work with skin equivalents (Goodarzi et al., 2018), or to work with *in vivo*, translucent model organisms more amenable to imaging. Bring on zebrafish or *Drosophila* (with their embryos, their larval imaginal discs, or later-stage pupae) and now it becomes possible to collect large datasets from high-resolution wound epithelial movies and to subject these to quantitative probing and mathematical modeling.

A recent study in the imaginal discs of *Drosophila* larvae used a vertex model (which represents epithelial cells as polygons with vertices) to simulate aspects of wound re-epithelialization and this indicated that myosin-mediated tissue fluidity, which enables cells to slip past each other, is critical for healing (Tetley et al., 2019). The model utilized an energy function with terms depending on cell area and line tension in the cell boundaries so that the forces acting on each vertex could be computed for each moment in time. In the computer simulation, if this tissue fluidity and thus cell shuffling were restricted by increasing the line tension, healing was significantly hindered. This inspired a real experiment to test the mathematical prediction which was achieved by forcing the expression of Rok RNAi in epidermal cells which successfully halted shuffling and did exactly as suggested by the simulation; it halted repair (Tetley et al., 2019), thus reinforcing the notion that, while leading edge epidermal cells are the powerhouses that drive re-epithelialization, cells further back must also proactively participate to release tissue tension and enable the wound to close (Razzell et al., 2014; Nunan et al., 2015).

**Figure 3:**
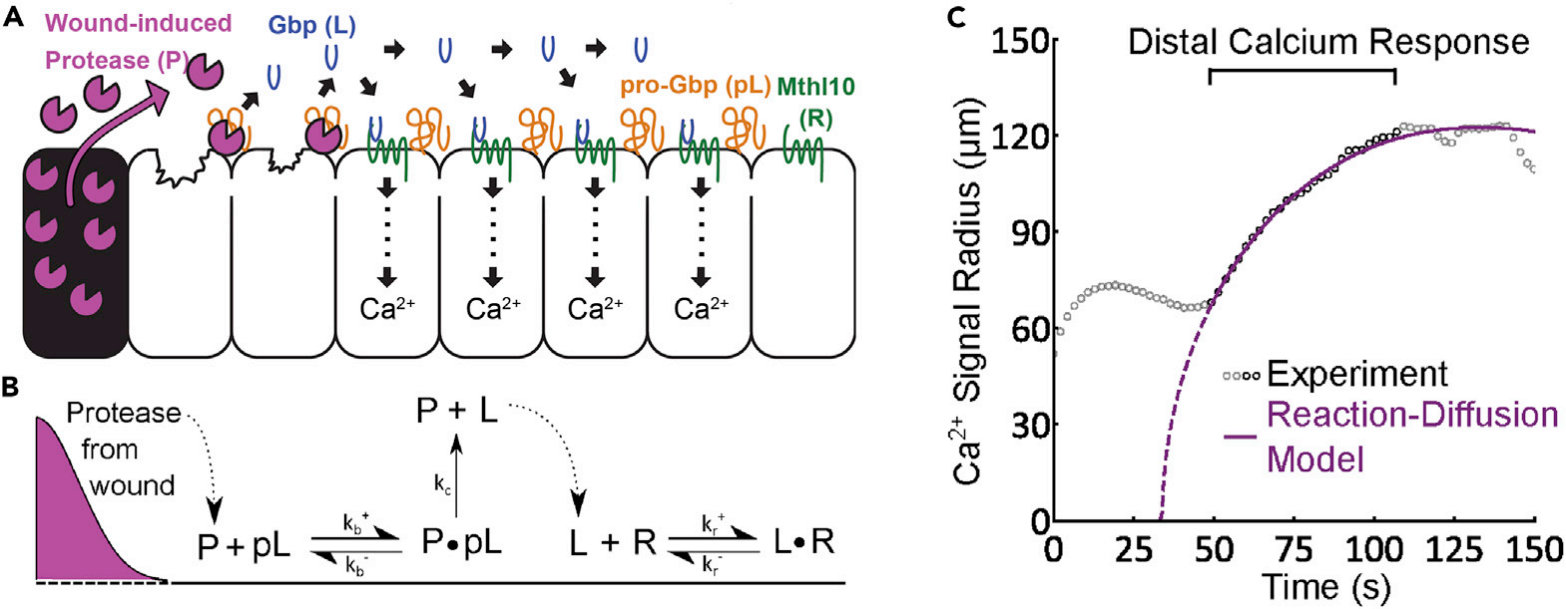
Modeling the wound-induced calcium wave in the *Drosophila* notum (A) The Hutson and Page-McCaw lab’s study utilized a diffusion-reaction model to better understand how the wound calcium signal is transduced. The model begins with the release of protease from lysed cells in the wound. This protease diffuses and can cleave pro-Gbp leading to the release and diffusion of Gbps. Gbps binds to Mthl10 leading to intracellular calcium release. (B) Resulting reaction-diffusion model. (C) The output of the model in purple compared with the experimental data (O’Connor et al., 2021).

If cells behind those at the immediate edge (with membrane ruptures), are to sense the wound and act in the ways described above, it must be that the damage signal is somehow transduced through or along the epithelial sheet adjacent to the wound. Several studies in *Drosophila* and other model organisms have shown how a calcium wave spreads from the wound edge outwards and is the first “damage” signal (Xu et al., 2012; Razzell et al., 2013). This calcium wave has been shown to, in part, be dependent on gap junctional communication between cells (Razzell et al., 2013), but a recent mathematics-biology study has shown that signal transduction is also mediated via proteases leaching from lysed wound cells that cleave and convert extracellular pro-peptides into activated Growth blocking peptides (GBPs), which in turn lead to the intracellular release of Ca^2+^ through binding to Methusela-like 10 (Mthl10) GPCRs in adjacent epithelial cells (Figure 3 and O’Connor et al., 2021). To model how this Ca^2+^ wave propagated, the authors used a complex reaction-diffusion model incorporating six components of the known signaling cascade including GBPs and Mthl10. Just as their experimental data had indicated, their model exhibited an increased effective diffusion of the calcium signal (and a shorter time delay), in larger wounds. Computational modeling also predicted that reducing the initial levels of Mthl10 would prevent triggering of the calcium response. When tested *in vivo*, the experiment matched this prediction, thus strongly supporting the notion that proteolytic cleavage of GBPs is, indeed, one way in which the Ca^2+^ wave is propagated (O’Connor et al., 2021).

### Modeling wound inflammation

Another key cell behavior integral to the repair process is the recruitment of innate immune (inflammatory) cells to the wound. Again, as for re-epithelialization, it has been helpful to be able to live image the dynamics of wound inflammation, which is possible but incredibly challenging in opaque mammalian tissues (Phillipson and Kubes, 2019), but has been made considerably easier through studies in the genetically tractable and translucent model organisms, *Drosophila* and zebrafish (Wood and Martin, 2017; LeBert and Huttenlocher, 2014). Of course, traditional knock-out (KO) studies in mouse have revealed some of the key attractants that draw immune cells to a wound (Phillipson and Kubes, 2019), but we still don’t know the full list of “damage” attractants, and for those we do know, we can only crudely visualize how a small number of them spread through tissues in those instances where there are genetic reporters or dyes to track them (Niethammer et al., 2009). It is also unclear how immune cells integrate all of these signals, in parallel and in series, and subsequently, how the inflammatory response eventually resolves after the wound is healed.

**Figure 4:**
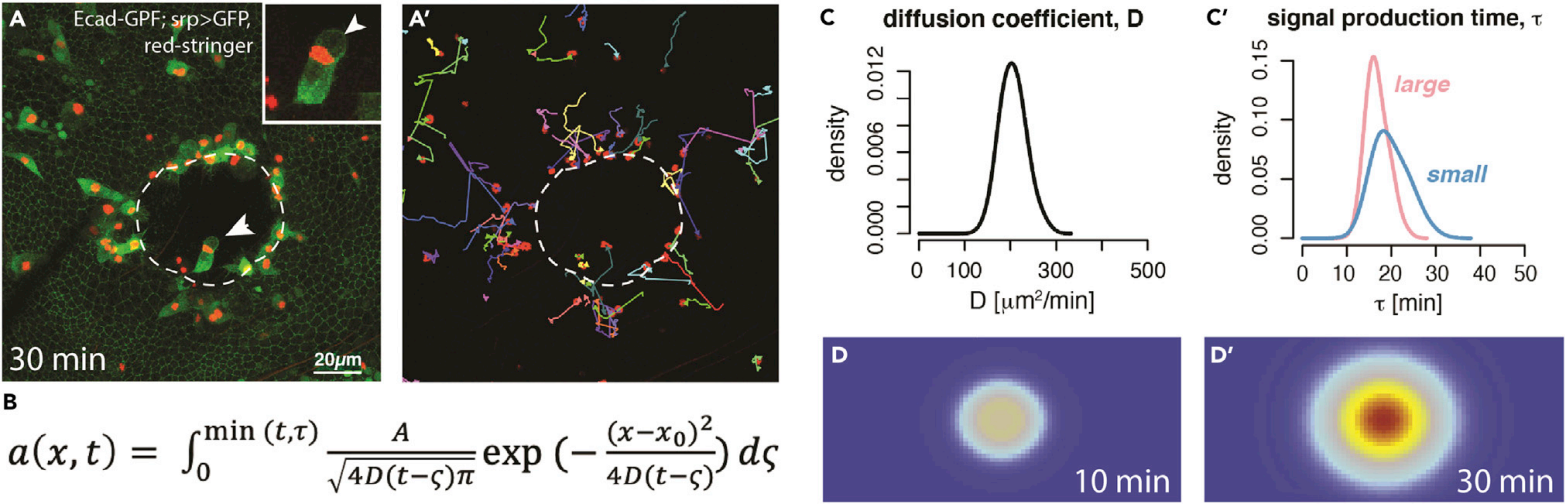
Modeling of the wound inflammatory response in the *Drosophila* pupal wing (A) Still image from a movie of a wound (white dashed line) made to the Drosophila pupal wing. Macrophages (green cytoplasm with red stingerRFP nuclei) are attracted to the wound by, as yet, unknown damage signals. a’ – tracks in a. (B) A diffusion equation with a fixed time period models the source and movement of the signal. Parameters of the equation can be estimated using Bayesian inference. a-concentration of attractant, D – diffusion coefficient, τ – signal production time. (C-C’ and D-D’) A simulation of wound chemoattractant using the parameters after 10mins and 30mins whereby warmer colors indicate a higher concentration (adapted with permission from Weavers et al., 2016).

In a recent study, we addressed some of these questions by applying Bayesian inference to interrogate large movie datasets of wounds made in *Drosophila* pupae that are translucent and thus enable us to capture the tracks of fluorescently tagged fly innate immune cells (termed hemocytes) as they respond to a laser wound (Weavers et al., 2016). We hypothesized that if we could “measure” the time difference between when the macrophages nearest the wound responded to the signal and when those further back did, then, much like determining how far away a lightning storm is by counting the seconds between the lightning strike and the subsequent clap of thunder, we might be able to determine the diffusion coefficient of the rate-limiting chemoattractant. And, indeed, with a large number of movies of unwounded versus wounded pupal wings (resulting in thousands of automated macrophage tracks), this data was fed into the Bayesian model in order to tune the optimal distribution of parameters in the model. Key biology parameters incorporated in the model included the diffusion constant, signal production time, and the amount of chemoattractant. This mathematical approach allowed us to detect a graded temporal response in the altered “bias” of macrophages to the wound dependent on the initial position relative to the wound, and to show that its diffusion coefficient was likely to be in the region of 200 μm^2^/min (Figure 4); this is about what one might expect for a growth factor or other large biomolecule, and much slower than expected for small molecules such as H_2_O_2_ and ATP (with predicted diffusion coefficients of 18000 *μm*^*2*^*/min* and 84,000 *μm*^*2*^*/min,* respectively, in water—Milo and Phillips, 2015), that have been shown experimentally to be important permissive damage attractant signals at the wound site (Moreira et al., 2010; Razzell et al., 2013). So, whilst mathematical analysis and computer simulation do-not reveal what the attractant is, it is able to say what it probably is not. As the bias response of macrophages tended to increase in a linear fashion as the diameter of wounds increased, rather than being proportional to increasing wound area, our simulation also suggested that the source of the attractant signal was the wound margin rather than the wound area *per se*. And it also suggested that whatever the size of the wound, new signal was produced for only 30 min. However, a second wound did not distract macrophages from the first wound until more than 3 h had elapsed, suggesting that receptors to the wound attractant might have been desensitized; this biology observation married to the mathematically derived diffusion coefficient strongly hints that the attractant could act via a GPCR receptor, thus potentially narrowing down our search considerably (Weavers et al., 2016). In an attempt to generate large enough wounds that they might release sufficient chemoattractant to saturate immune cell receptors before the cells had reached the wound, we made the serendipitous finding that some of these large wounds were delayed in healing. This wound chronicity was associated with a significantly altered dynamic behavior of the immune cells which exhibited significantly more “random walk” behavior at early timepoints post wounding that we were able to quantify precisely. This hints at a potential novel prognostic indicator of wound chronicity to complement other potential predictors such as transcriptomic signatures (Bosanquet et al., 2019).

Of course, no mathematical model is perfect, but is rather a controlled approximation of reality; another clear weakness here is that *Drosophila* pupal wounds are miniature, and so, by definition, cannot perfectly model a larger more complex wound in, for example, patient skin where immune cells will be recruited from a greater distance through more complex terrain. Almost certainly, it will turn out to be the case that our presumption of a single attractant is a gross simplification. But this reductionist approach in flies sets the scene for layering on additional wound attractants that may work in parallel or series to draw immune cells, from source (sometimes even from vessels (Thuma et al., 2018)) to the damage site. Ultimately, of course, our aspiration must be to gain high-resolution imaging data from human patients and integrate mathematical modeling in the analysis of this data in order to learn more about clinical wound inflammation, and why it can go wrong than can be revealed by the biology alone.

## Other important wound questions that might benefit from some mathematical modeling input

Whilst re-epithelialization and wound inflammation are undoubtedly key aspects of the repair response that have recently become amenable to mathematical modeling, other components of healing might also be ripe for similar investigation:

**Wound angiogenesis is a pivotal aspect of the repair process.** The massively increased metabolic demands of healing tissue require increased oxygen and nutrients and, consequently, the cutaneous vascular plexus undergoes an episode of massive branch sprouting in order to supply these needs. The importance of angiogenesis in wound healing is reflected clinically in the observation that chronic wounds often have very poor vascularity, whereas overhealing, keloid wounds are generally excessively vascularized (Martin and Gurevich, 2021). After the wound has repaired, the excess vasculature needs to trimmed back to normal. Whilst re-epithelialization can be envisaged almost as a 2-D process happening in one plane, wound angiogenesis is more complex and operates in 3-D. Vessel sprouting in wound granulation tissue is known to be regulated by a series of inhibitory and positive (including a growth factor, vascular endothelial factor, VEGF) molecules, delivered by inflammatory cells (Gurevich et al., 2018), but the details of this process and its resolution, and how it can fail (as in a chronic wound), are still poorly understood.

There have been previous attempts to model wound angiogenesis utilizing reaction-transport mathematical models (Flegg et al., 2009; Schugart et al., 2008). These models use different variables and incorporate key parameters including inflammatory cell density, growth factor concentrations, capillary tip density, blood vessel density, and oxygen concentration. By analyzing the interactions of these variables in space, modelers have been able to produce a qualitative match to published experimental data (Flegg et al., 2009; Schugart et al., 2008). What they miss, however, as mentioned above, is the role of mechanics and how it feeds back into chemistry which is required for a truly quantitative and predictive model.

Many of us in the wound healing community believe that there are key parallels between wound healing and cancer (MacCarthy-Morrogh and Martin, 2020), and tumor angiogenesis has been extensively mathematically modeled using discrete forms of the continuous model. One such study undertook a detailed analysis of the blood vessel network including loop formation and sprouting density and length. The localized nature of such a model means it can include local mechanics, for example, the interactions between the extracellular matrix components (Chaplain, 2000). Another study modeled vasculogenesis using an alternative combination of continuous (reaction-diffusion) and discrete (Cellular Potts) models (Scianna et al., 2011). This allowed them to include intracellular biochemistry and local biophysics in their simulations of vasculogenesis which, in turn, matched well with *in vitro* knockdown experiments.

Applying these mathematical approaches to produce models that produce similar networks but for a wound (rather than cancer) scenario would enable predictive studies of wound angiogenesis. This is clearly an area where mathematicians and physicists should work together with biologists, preferably biologists who work with a model organism where this process can be live imaged and which is genetically tractable, so that predictions from modeling can be tested to enable an iterative process which would further the understanding of both the maths and biology of this problem. Indeed, now there is considerably more molecular information about wound angiogenesis to feed into the models, and even the complexity of these interactions in 3-D will be more tractable using mathematical approaches.

**Collagen deposition to generate a scar** is a longstanding, and still unresolved problem for clinicians and wound biologists. There are still no science-driven medicines available for clinicians to inhibit the scarring process. But we now have many insights from *in vivo* studies in mice and zebrafish as to how scar collagen is laid down and we know that scarring is, to a large degree, regulated, just as angiogenesis, by the wound inflammatory response. There are now even opportunities to live image collagen deposition at the wound site utilizing collagen reporter lines in zebrafish (Morris et al., 2018). Precisely how collagen is deposited and subsequently cross-linked into aligned “scar” bundles feels like a problem at the interface of biology and mathematical modeling. Indeed, this is a problem where modeling was previously attempted by drawing together parameters including fibroblast “concentration,” trajectory, and responsiveness to chemoattractant gradient (McDougall et al., 2006). These early modelers used a combination of discrete (for fibroblast paths) and continua (for collagen and chemoattractant) variables to determine how these parameters interact and feedback on one other— at a time when very little of the *in vivo* biology had yet been characterized. Now we know considerably more about biology and even have numbers to feed into some of the key parameters where we had previously only guestimates, (eg Morris et al., 2018; Canty and Kadler, 2005) so this might be an ideal time to revisit the collagen deposition/scarring problem using mathematical modeling approaches.

**Regeneration of appendages after tissue repair** - hairs and sweat glands are not regenerated at a wound site unless (at least) the stumps of these structures are left behind by the tissue damage (Box 4). So, for example, in a deep burn wound, when the epidermis eventually covers over the wound there will be no hairs or sweat glands at the scar site, even if a clinician provides a split skin graft as is likely if the lesion is extensive. Because the development of evenly spaced hairs across the skin during embryogenesis has been shown to be owing to an interplay of notch/delta positive versus inhibitory signals, in ways modeled using Turing-style reaction-diffusion equations (Lewis, 1998), it is likely that the same would need to be recapitulated if we are ever to regenerate appendages in a healing wound. One remarkable, but somewhat confusing, observation in mouse wound studies, has revealed that islands of regenerating hairs can arise in the center of some wounds over a certain size (Ito et al., 2007). A mathematical model incorporating mechano-chemical feedback may explain this phenomenon and flag up possible candidates for signaling mechanisms that might trigger hair regeneration where previously there was none.

Box 4 **Regeneration of skin appendages**

Skin appendages, hairs, and sweat and sebaceous glands, can repair completely after tissue damage, so long as some remnant of them is left in the exposed deep dermis. But if all vestige of them has been lost or destroyed, for example, following a deep skin burn, then they do not regenerate except in exceptional, poorly understood circumstances. Appendage stumps are also a good source of epidermal cells for repairing interfollicular epidermis so that repair is not entirely dependent on marginal epidermal wound edge cells.

## The future is bright for mathematics-physics-biology marriages in wound-healing biology

The two earlier vignettes of re-epithelialization and wound inflammation we describe above, show how mathematical and physics modeling approaches can be highly informative in providing us with a better understanding of the wound healing process. Clearly, the right biology questions need to be asked and the appropriate mathematics and physics brought into play in order to offer useful insights. For this reason, and for mathematics and biology to be additive (maybe even more than additive) rather than simply complementary, there needs to be effective integration between the mathematical biologists and the wet lab experimentalists in order for the iterative process of maths simulation and predictive experimentation to work smoothly. It could be argued that inflammation and re-epithelialization are somewhat low hanging fruit because they can be observed in 2D, and there is no doubt that some of the additional wound healing “wants” that we outline above, will be complicated by their more 3D nature and/or by not being so amenable to study in simple, translucent and genetically tractable model organisms.

So, what are the major challenges to this interdisciplinary research and how might we overcome them? In our experience, nothing beats sitting down and discussing at length the fundamental biology (and clinical) questions to determine what might be the best mathematical tools to address the problem; clearly, lots of compromise is required here because sometimes big biology questions only require basic mathematics and physics and vice versa. However, what is already clear is that current and future advancements in computer vision and deep learning offer even greater opportunities to extract information quickly from even the largest biological datasets, consistently and with high accuracy. This is particularly important when dealing with the large “movie” datasets available with current imaging protocols, as tracking and analyzing data by hand is time-consuming and prone to human error. Combining these approaches will enable modeling of increasingly complex systems as is the norm in wound healing biology, and so it is not unreasonable to hope for bigger and better from the mathematics-physics-biology wound interface in the near future. Indeed, a longer-term aspiration for wound researchers will be the inclusion and analysis of clinical data which will, by definition, be much more complex than for the translucent model organisms which are currently most amenable to maths/biology studies.
